# Prevalence and influencing factors of oral frailty in cancer patients: a systematic review and meta-analysis

**DOI:** 10.3389/fpubh.2026.1859081

**Published:** 2026-08-24

**Authors:** Wenxiao Liu, Meng Fang, Yukai Luo, Tianshu Mei, Ping Huang

**Affiliations:** 1 Department of Emergency, Nanjing Drum Tower Hospital, Affiliated Hospital of Medical School, Nanjing University, Nanjing, Jiangsu, China; 2 School of Nursing, Nanjing University of Chinese Medicine, Nanjing, China

**Keywords:** cancer, influencing factors, meta-analysis, oral frailty, prevalence

## Abstract

**Introduction:**

This study systematically evaluated the prevalence of oral frailty and its associated influencing factors among patients with cancer through a comprehensive systematic review and meta-analysis.

**Methods:**

A systematic search was conducted across nine electronic databases up to January 13, 2026, and 13 studies involving 3,524 participants were ultimately included.

**Results:**

The pooled analysis demonstrated that the prevalence of oral frailty in patients with cancer was 52% (95% CI: 46%–59%), indicating a substantial disease burden in this population. Several factors were identified as associated with oral frailty, including denture use (OR = 15.09, 95% CI: 6.78–33.58), poor oral health (OR = 4.70, 95% CI: 2.13–10.39), physical frailty (OR = 3.63, 95% CI: 2.40–5.50), smoking (OR = 3.53, 95% CI: 2.34–5.31), malnutrition (OR = 3.40, 95% CI: 2.01-5.75), xerostomia (OR = 3.46, 95% CI: 2.11–5.68), and radiotherapy (OR = 2.62, 95% CI: 1.67–4.11). Sensitivity analysis confirmed the robustness of the results, and Egger's test indicated no significant publication bias (*P* > 0.05).

**Discussion:**

These findings suggest that oral frailty is relatively common among patients with cancer and is associated with several identifiable factors.

**Systematic review registration:**

https://www.crd.york.ac.uk/PROSPERO/, identifier: CRD420261281120.

## Introduction

In recent years, with the acceleration of global population aging and the continuous rise in cancer incidence, cancer has emerged as one of the most critical public health challenges threatening human health worldwide. According to the World Health Organization, cancer-related mortality constitutes a leading cause of death globally. The prolonged course of cancer treatment, including chemotherapy and radiotherapy, not only affects patients' survival outcomes but also substantially impairs their quality of life (1). In this context, identifying key factors that influence functional status and prognosis in cancer patients has become a major focus of contemporary clinical research.

Oral health, as an integral component of overall health, is particularly critical in patients with cancer. In recent years, the emerging concept of oral frailty has gained increasing attention. It is generally characterized by a spectrum of functional declines, including reduced masticatory function, dysphagia, xerostomia, and deterioration of oral hygiene status. Accumulating evidence suggests that oral frailty is not only closely associated with malnutrition, physical frailty, and increased mortality risk in older adults, but may also exacerbate disease progression by impairing dietary intake and immune function (2). In patients with cancer, the risk of developing oral frailty is substantially elevated, which is closely related to both the malignancy itself and its treatment modalities. In particular, therapies such as radiotherapy and chemotherapy can induce oral mucosal injury and salivary gland dysfunction, thereby further contributing to the onset and progression of oral frailty (3).

Current evidence suggests that the prevalence of oral frailty among patients with cancer is relatively high and may be influenced by multiple factors, including smoking, xerostomia, denture use, malnutrition, and physical frailty (4, 5). For instance, xerostomia can impair the self-cleansing capacity of the oral cavity by reducing salivary secretion, thereby increasing the risk of bacterial colonization (6). In addition, improper denture use may lead to oral mucosal injury and chronic inflammatory responses, further exacerbating the decline in oral function (7). Moreover, treatment-related adverse effects, particularly those associated with radiotherapy, have also been identified as important contributors to the development of oral frailty (8). However, substantial heterogeneity exists across studies in terms of sample size, study design, and assessment tools, resulting in inconsistent findings and thereby limiting the generalizability of the available evidence.

Despite increasing research on oral frailty in cancer patients, evidence synthesizing its prevalence and associated factors remains limited. Existing studies are predominantly cross-sectional and heterogeneous, resulting in inconsistent findings and unclear overall effects of risk factors. Therefore, a systematic review and meta-analysis was conducted to provide a comprehensive evaluation. This study pooled the prevalence of oral frailty and examined its influencing factors to support early risk identification and guide targeted clinical interventions.

## Methods

This systematic review was reported in accordance with the Preferred Reporting Items for Systematic Reviews and Meta-Analyses (PRISMA) guidelines. The study was registered at the PROSPERO with registration number CRD420261281120 (https://www.crd.york.ac.uk/PROSPERO/view/CRD420261281120).

### Search strategy

We searched PubMed, Web of Science, Embase, the Cochrane Library, CINAHL, VIP, Sinomed, Wanfang, and CNKI for relevant studies published up to January 13, 2026. The search strategy employed a combination of controlled vocabulary (e.g., MeSH terms) and free-text terms with Boolean operators to ensure comprehensive retrieval. The search strategy included terms related to “oral function,” “oral weakness,” “oral frailty,” “cancer.” See Supplementary file 1 for details.

### Inclusion and exclusion criteria

The criteria for inclusion of a study in the systematic review were as follows: (1) they enrolled participants with a cancer diagnosis; (2) they employed a clearly defined conceptualization or validated assessment tool for oral frailty; (3) they reported oral frailty as an outcome variable; (4) they utilized a cross-sectional, cohort, or case-control design; and (5) they achieved a quality appraisal score greater than 4. Studies were excluded if they met any of the following criteria: (1) they included participants diagnosed with oral cancer; (2) they were reviews, conference abstracts, or opinion pieces (e.g., commentaries); (3) relevant data were unavailable for extraction; or (4) they were published in languages other than English or Chinese. The reason for excluding patients with oral cancer from this study was that the primary tumor itself has a direct impact on oral structure and function, which may confound the assessment of oral frailty. This study focused on patients with non-head and neck cancers to explore the indirect effects of cancer treatment on oral function.

### Assessment of risk of bias

Two reviewers independently used the evaluation scale developed by the Agency for Healthcare Research and Quality (AHRQ) (9) to assess the quality of the literature. This tool comprises 11 items, with each item rated as “Yes,” “No,” or “Unclear.” The maximum possible score is 11 points, with scores of 0–3 indicating low quality, 4–7 indicating moderate quality, and 8–11 indicating high quality. Any discrepancies were resolved by consultation with a third reviewer until consensus was reached. See Supplementary file 2 for details.

### Data extraction

Two investigators independently evaluated the titles, abstracts, and full texts of the retrieved literature to determine their eligibility for inclusion. Studies that met the predefined inclusion and exclusion criteria were included in this study. Two reviewers independently extracted study characteristics, including authors, publication year, country, sample size, study design, number of study centers, study population, participant age, prevalence, influencing factors, diagnostic criteria, and odds ratio (OR). All extracted data were managed using Excel. During the literature screening and data extraction processes, any disagreements between the two investigators were resolved through discussion with a third investigator until consensus was reached.

### Data analysis and synthesis

For each influencing factor, effect sizes were extracted from the original literature of included studies as odds ratios (ORs) with their corresponding 95% confidence intervals (95% CIs). Adjusted ORs (ORs) derived from multivariable analyses that controlled for potential confounders were preferentially adopted. Between-study heterogeneity was assessed using the Cochrane Q test and the I^2^ statistic. The model for effect size pooling was selected based on heterogeneity test results: a fixed-effects model (inverse-variance method, IV) was employed when heterogeneity was low (I^2^ < 50% and *P* ≥ 0.1), whereas a random-effects model (DerSimonian-Laird method, D-L) was applied in the presence of significant heterogeneity (I^2^ ≥ 50% and *P* < 0.1). The robustness of the pooled estimates was evaluated using a leave-one-out sensitivity analysis. Publication bias was assessed qualitatively using funnel plots for each influencing factor. When the number of included studies was ≥ 10, Egger's linear regression test was further conducted for quantitative assessment of publication bias.

## Results

**Figure 1:**
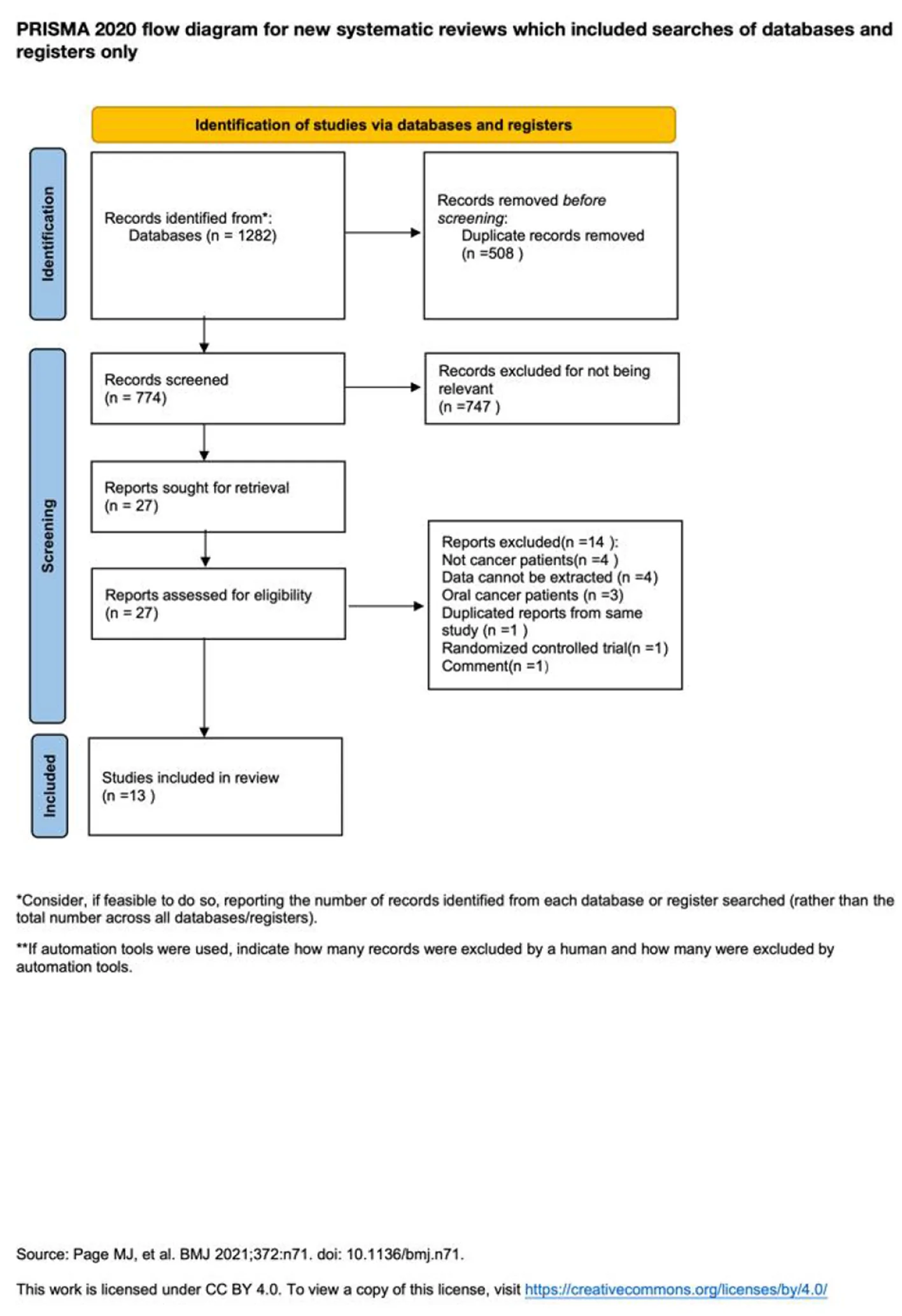
Flow diagramfor literature selection.

The initial literature search yielded 1,282 records. After the removal of duplicates using EndNote software, 774 records remained. Following title and abstract screening, 747 irrelevant records were excluded, leaving 27 articles for full-text review. During full-text assessment, a further 14 articles were excluded due to inconsistency with the research topic. Ultimately, 13 studies met the inclusion criteria and were included in this review, comprising 5 studies published in Chinese and 8 in English. The overall process of literature screening is shown in Figure 1.

### Article characteristics

A total of 13 cross-sectional studies (4, 5, 10–20) comprising 3,524 participants were included in this review, published between 2023 and 2026. Among these, 10 studies were conducted in China and 3 in Japan. In the quality assessment of the included studies, 4 were rated as high quality and 9 as moderate quality. Three distinct diagnostic criteria were employed to assess oral frailty in cancer patients across the included studies. Of the 13 studies, 10 reported on factors associated with oral frailty, and all 13 reported prevalence estimates. Sample sizes ranged from 60 to 555 participants, with reported prevalence of oral frailty among cancer patients ranging from 28.2% to 64.3%. Details are provided in Table 1.

**Table 1:** Study characteristics.

Study	Year	Country	Sample size	Type of study	Subject	Age	Prevalence of oral frailty	Risk factors	Diagnostic criteria	Number of research centers	AHRQ
Katsushima et al. (10)	2026	Japan	67	Cross-sectional study	Patients with lung cancer	73	46.27%	/	OF-5	Single-center study	High quality
Li et al. (4)	2025	China	368	Cross-sectional study	Patients with cancer undergoing chemotherapy	60.60	57.58%	②, ③, ④, ⑤, ⑥, ⑦, ⑧	OFI-8	Multicenter study	High quality
Teranishi et al. (11)	2023	Japan	60	Cross-sectional study	Patients with cancer who underwent distal gastrectomy	/	48.33%	/	Functional tooth units	Single-center study	Moderate quality
Wang et al. (12)	2025	China	270	Cross-sectional study	Older cancer patients	69.8	57.70%	⑤, ⑥, ⑨, ⑩, ⑪, ⑫	OFI-8	Multicenter study	High quality
Shen et al. (13)	2025	China	139	Cross-sectional study	Patients diagnosed with thoracic tumors	/	39.57%	/	OFI-8	Single-center study	Moderate quality
Shimizu et al. (14)	2025	Japan	181	Cross-sectional study	Patients with gastrointestinal cancers	68.0	28.20%	⑨, ⑬, ⑭, ⑮	OFI-8	Single-center study	Moderate quality
Liu et al. (15)	2025	China	180	Cross-sectional study	Patients with gynecologic tumors	/	39.44%	⑨, ⑯	OFI-8	Single-center study	Moderate quality
Liu et al. (16)	2025	China	431	Cross-sectional study	Lung cancer patients undergoing chemotherapy	/	58.93%	①, ②, ⑰, ⑱, ⑲	OFI-8	Multicenter study	Moderate quality
Lu et al. (17)	2025	China	406	Cross-sectional study	Hospitalized older adult cancer patients	69.0	63.50%	①, ④, ⑪, ⑫, ⑳, ㉑, ㉒	OFI-8	Multicenter study	Moderate quality
Su et al. (18)	2025	China	269	Cross-sectional study	Patients with gastrointestinal tumors	/	63.56%	⑤, ⑩, ⑪, ⑫, ㉓, ㉕	OFI-8	Single-center study	Moderate quality
Li et al. (19)	2024	China	207	Cross-sectional study	Hospitalized cancer patients	63.61	64.30%	①, ②, ⑥, ⑨, ⑩, ⑪, ㉔	OFI-8	Single-center study	Moderate quality
Bu et al. (20)	2025	China	206	Cross-sectional study	Patients with lymphoma undergoing chemotherapy	57.8	44.66%	①, ⑦, ⑧, ㉕, ㉖	OFI-8	Multicenter study	Moderate quality
Lv et al. (5)	2026	China	555	Cross-sectional study	Older adult patients with esophageal cancer	/	49.19%	④, ⑨, ⑩, ㉗	OFI-8	Multicenter study	High quality

Explanation: ① Age; ② Educational level; ③ Disease duration; ④ Radiation therapy; ⑤ Xerostomia; ⑥ Denture use; ⑦ Chemotherapy-induced taste alteration; ⑧ Family functioning; ⑨ Smoking; ⑩ Physical frailty; ⑪ Poor oral health; ⑫ Malnutrition; ⑬ Hemoglobin; ⑭ Albumin; ⑮ Neoadjuvant therapy; ⑯ Dietary diversity score; ⑰ Brain metastasis; ⑱ Lactate dehydrogenase; ⑲ C-reactive protein; ⑳ Oral mucositis; ㉑ Grip strength; ㉒ Low oral health self-efficacy; ㉓ Nasogastric feeding; ㉔ History of chemotherapy; ㉕ Depression; ㉖ Insomnia; ㉗ Tumor staging; OF-5, The 5-item oral frailty checklist; OFI-8, The oral frailty index-8.

### Meta-analysis of the overall prevalence of oral frailty in cancer patients

**Figure 2:**
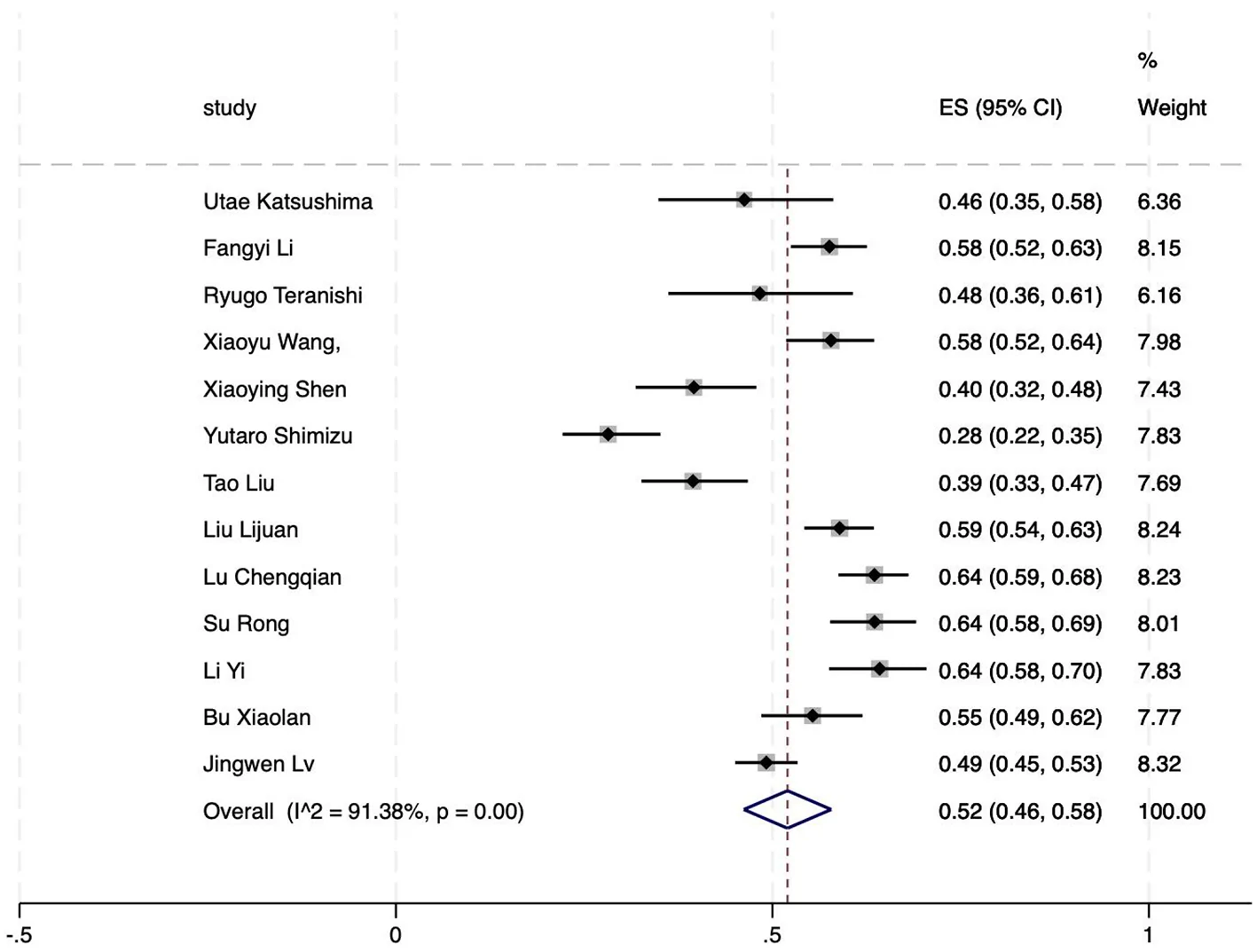
Forest plot of the prevalence of oral frailty.

A total of 13 studies (*n* = 3,524 patients) were included in the meta-analysis of prevalence. Using a random-effects model, the pooled prevalence of oral frailty in patients with cancer was 52% (95% CI: 46%−58%). However, substantial heterogeneity was observed among the studies (I^2^ = 91.4%, *P* < 0.001). The results showed a statistically significant difference between subgroups stratified by sample size (*P* < 0.001), indicating that sample size may be a source of heterogeneity. In contrast, subgroup analyses based on sex, country, assessment tool, number of study centers, TNM stage and cancer type did not show any statistically significant between-subgroup differences (*P* > 0.05), suggesting that these factors are unlikely to be major sources of heterogeneity. The forest plot of the meta-analysis of oral frailty prevalence in patients with cancer is presented in Figure 2, and the results of the subgroup analyses are shown in Table 2.

**Table 2:** Subgroup analysis of the prevalence of oral frailty symptoms in cancer patients.

Subgroups	No. of studies	Sample	Oral frailty	Low	High	Prevalence	I^2^	*P*
Gender								
Male	5	746	409	0.50	0.72	61%	81.5%	< 0.001
Female	5	418	250	0.43	0.74	58%	94.5%	< 0.001
Country								
China	10	3,026	1,694	0.50	0.60	55%	87.4%	< 0.001
Japan	3	308	111	0.26	0.55	40%	83.1%	0.003
Sample size								
*n* < 200	5	627	237	0.32	0.46	39%	69.7%	0.010
200 ≤ *n* < 400	5	1,315	783	0.56	0.63	60%	36.2%	0.180
*n* ≥ 400	3	1,392	785	0.49	0.66	57%	90.8%	< 0.001
Tools								
Oral frailty index-8	11	3,207	1,745	0.46	0.59	53%	92.7%	< 0.001
Other	2	127	60	0.39	0.56	47%	0	0.816
Research centers								
Single-center	7	1,103	541	0.35	0.59	47%	93.8%	< 0.001
Multicenter	6	2,231	1,264	0.53	0.61	57%	77.3%	< 0.001
TNM								
I	4	121	34	0.12	0.6	36%	81.4%	< 0.001
II-III	5	531	252	0.42	0.63	53%	83.4%	< 0.001
IV	4	518	244	0.55	0.65	60%	13.4%	0.326
Cancer type								
Lung cancer	2	498	285	0.53	0.62	57%	/	/
Gastrointestinal tumors	4	1,065	524	0.45	0.53	49%	95.27%	< 0.001

Detailed results of the subgroup analyses of the prevalence of oral frailty in patients with cancer are presented in Supplementary file 3.

### Meta-analysis of factors associated with oral frailty in cancer patients

Two studies reported an association between denture use and oral frailty. With low heterogeneity (I^2^ < 50%), a fixed-effects model was applied. The results indicated that denture use was associated with oral frailty (OR = 15.09, 95% CI: 6.78–33.58, *P* < 0.001).

Four studies associated poor oral health status with oral frailty. Given the presence of moderate heterogeneity (I^2^ = 54%), a random-effects model was used. The results indicated that poor oral health status was associated with oral frailty (OR = 4.70, 95% CI: 2.13–10.39, *P* < 0.001).

Four studies reported an association between physical frailty and oral frailty. With low heterogeneity (I^2^ < 50%), a fixed-effects model was applied. The results indicated that physical frailty was associated with oral frailty (OR = 3.63, 95% CI: 2.40–5.50, *P* < 0.001).

Four studies reported an association between smoking and oral frailty. Among them, the study by Liu et al. (15) included 100% female patients with gynecological cancer, whereas the other three studies included mixed sexes. Given that the association between smoking and oral health may differ by sex, this study was excluded. After exclusion, heterogeneity decreased substantially (I^2^ < 50%), and a fixed-effects model was applied. The pooled results indicated that smoking was associated with oral frailty in patients with cancer (OR = 3.53, 95% CI: 2.34–5.31, *P* < 0.001).

Three studies reported an association between malnutrition and oral frailty. With low heterogeneity (I^2^ < 50%), a fixed-effects model was used. The results showed that malnutrition was associated with oral frailty (OR = 3.40, 95% CI: 2.01–5.75, *P* < 0.001).

Three studies reported an association between xerostomia and oral frailty. With low heterogeneity (I^2^ < 50%), a fixed-effects model was applied. The results indicated that xerostomia was associated with the risk of oral frailty (OR = 3.46, 95% CI: 2.11–5.68, *P* < 0.001).

Two studies reported an association between radiotherapy and oral frailty. With no heterogeneity (I^2^ = 0%), a fixed-effects model was used. The results indicated that radiotherapy was associated with oral frailty in patients with cancer (OR = 2.62, 95% CI: 1.67–4.11, *P* < 0.0001).

Meta-analyses were also conducted for depression and age; however, none of these factors reached statistical significance (*P* > 0.05).

Detailed results of the meta-analysis of influencing factors for oral frailty in patients with cancer are presented in Table 3 and Supplementary file 3.

**Table 3:** Risk factors for oral frailty in cancer patients.

Influencing factors	Number of studies	Estimation of combined			Effect model	Heterogeneity test	
		OR (95% CI)	Z	*P*		I^2^	*P*
Dentures	2	15.09 (6.78–33.58)	6.65	< 0.00001	Fixed	0	0.44
Poor oral health	4	4.70 (2.13–10.39)	3.83	0.0001	Random	54%	0.09
Physical frailty	4	3.63 (2.40–5.50)	6.09	< 0.00001	Fixed	31%	0.23
Smoking	3	3.53 (2.34–5.31)	6.04	< 0.00001	Fixed	0	0.84
Malnutrition	3	3.40 (2.01–5.75)	4.57	< 0.00001	Fixed	0%	0.86
Xerostomia	3	3.46 (2.11–5.68)	7.94	< 0.00001	Fixed	0%	0.39
Radiotherapy	2	2.62 (1.67–4.11)	4.21	< 0.00001	Fixed	0%	0.54
Chemotherapy-induced taste alteration	2	2.94 (0.25–34.4)	0.86	0.39	Random	84%	0.01
Depression	2	2.16 (0.78–6)	1.47	0.14	Random	74%	0.05
Family functioning	2	1.81 (0.98–3.36)	1.89	0.06	Random	96%	< 0.00001
Age	3	1.43 (0.94–2.15)	1.68	0.09	Random	71%	0.03

### Sensitivity and publication bias analysis

**Figure 3:**
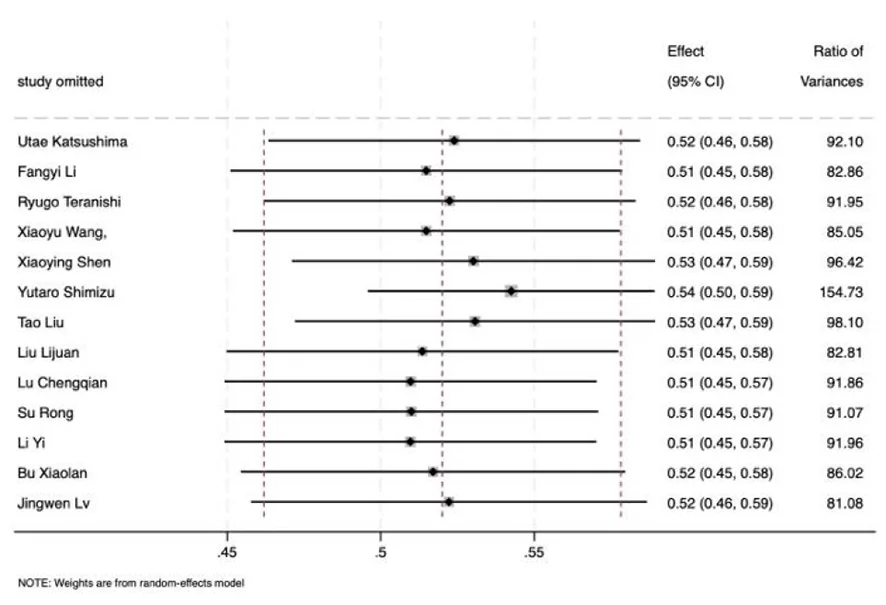
Sensitivity analysis of the prevalence of oral frailty among patients with cancer.

**Figure 4:**
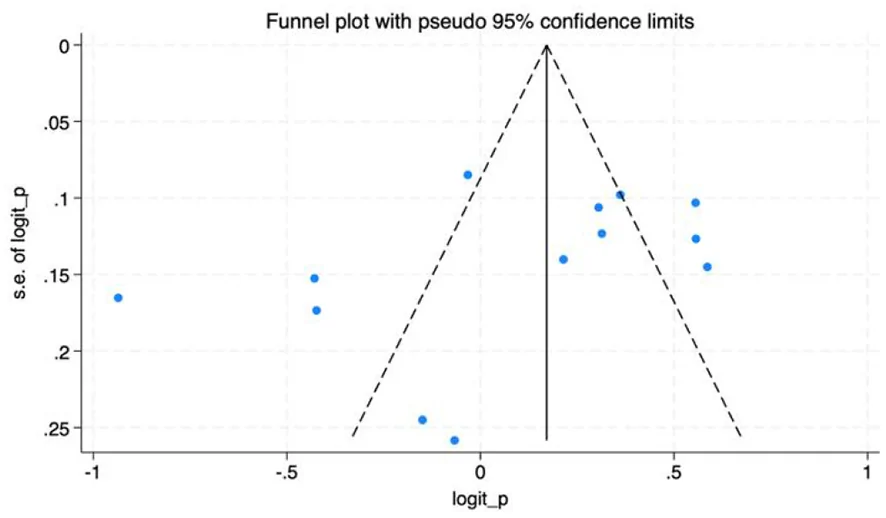
Funnel plot for publication bias

**Figure 5:**
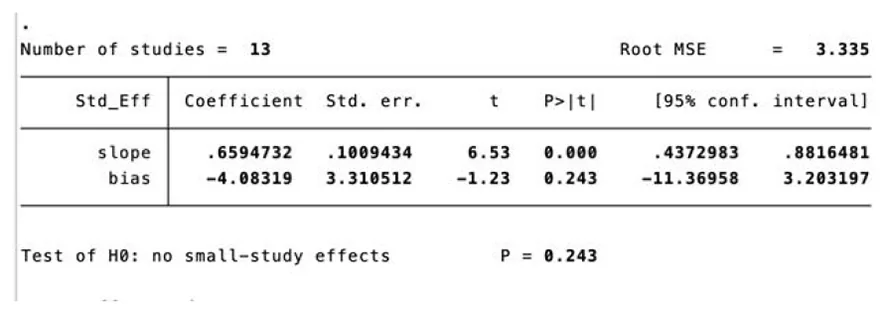
Egger's linear regression test for publication bias.

As shown in Figure 3, sensitivity analysis was conducted using a leave-one-out approach. The results demonstrated that, after excluding each study in turn, the pooled prevalence ranged from 51% to 54% without any change in direction, indicating that the findings were robust and stable. Although the study by Shimizu et al. (14) had a relatively greater influence on the overall estimate, its exclusion did not alter the overall conclusions. Publication bias was assessed using funnel plots and Egger's test. Despite the visual asymmetry observed in the funnel plot, Egger's test yielded a result of *P* = 0.243 (> 0.05), suggesting no significant publication bias. However, given the limited number of included studies and the substantial heterogeneity, these findings should be interpreted with caution. Detailed results are presented in Figures 4, 5.

## Discussion

This study included 13 studies involving a total of 3,524 patients with cancer. To the best of our knowledge, this is the first systematic review and meta-analysis to provide a quantitative synthesis of the prevalence and key determinants of oral frailty in this population. Due to the limited number of available studies, only two countries were represented: China (*n* = 10) and Japan (*n* = 3). This limited geographic representation may restrict the generalizability of the findings to broader populations. The results of this study showed that multiple factors were significantly associated with oral frailty in patients with cancer, including denture use (OR = 15.09), poor oral health status (OR = 4.70), physical frailty (OR = 3.63), smoking (OR = 3.53), malnutrition (OR = 3.40), xerostomia (OR = 3.46), and radiotherapy (OR = 2.62). The pooled prevalence of oral frailty in patients with cancer was 52% (95% CI: 0.46–0.58; I^2^ = 91.38%, *P* < 0.001). To further explore potential sources of heterogeneity, subgroup analyses were performed based on sex, country, sample size, assessment tool, number of study centers, cancer type, and disease stage. The results indicated that sample size might be a major source of heterogeneity.

### Prevalence of oral frailty in patients with cancer

The pooled prevalence observed in this study (52%) was numerically higher than that previously reported for the general older population (21), and for community-dwelling older adults (22), and was comparable to that reported for patients with diabetes mellitus (23).

Subgroup analysis revealed that the prevalence of oral frailty varied across different cancer types, although the between-group differences did not reach statistical significance. From a clinical perspective, this trend in differential prevalence may suggest the need for individualized oral function assessment strategies tailored to specific cancer types. From a biological perspective, variations in oral frailty prevalence across cancer types may be attributable to differences in disease stage and treatment modalities. Patients with early-stage tumors were primarily managed with surgery alone, with a relatively low proportion receiving chemoradiotherapy (24); consequently, the toxic effects of antineoplastic therapy on salivary glands, oral mucosa, and masticatory muscles were relatively limited. As the disease progressed to the intermediate stage, most patients required chemotherapy in addition to surgery, with some receiving adjuvant local radiotherapy (25), leading to the emergence of chemotherapy-related oral mucositis, taste disturbances, and systemic fatigue. In the advanced stage, patients were generally treated with intensive combined chemoradiotherapy regimens (26), which may cause sustained damage to salivary glands and disruption of the oral microenvironment. Furthermore, patients with advanced-stage cancer tended to exhibit poorer overall physical condition (27). Advanced malignancy is frequently accompanied by tumor-related consumption, cachexia, and severe malnutrition, which may result in systemic muscle loss extending to oral-related muscles such as the tongue, masseter, and swallowing muscles, potentially contributing to oral functional decline. Additionally, patients with advanced-stage cancer appeared to experience substantially higher levels of psychological distress, which could negatively affect their daily activity capacity and willingness to maintain oral hygiene (28).

Subgroup analysis revealed that the prevalence of oral frailty was higher in Chinese populations compared to Japanese populations. This disparity may be attributed to cultural differences and variations in healthcare systems. As a developed country, Japan has integrated dental care into its universal health coverage system with low out-of-pocket costs, which may facilitate better access to oral healthcare and contribute to the lower prevalence observed (29). Furthermore, Japan has implemented the “8020 Campaign,” an initiative aimed at ensuring that individuals retain at least 20 functional teeth by the age of 80. This public health campaign has promoted positive oral health behaviors among the Japanese population and contributed to improved dental function in older adults (30).

In this study, all included studies were cross-sectional in design. The quality assessment indicated that 4 studies were rated as high quality and 9 as moderate quality, with moderate-quality studies constituting the majority. Moderate-quality studies may have exhibited relatively insufficient standardization in enrollment criteria control, bias management, and indicator measurement, which could potentially contribute to variability in study findings. Small-sample studies might be more susceptible to sampling error and selection bias, possibly leading to an inflated estimation of outcome effect sizes. In contrast, large-sample studies appeared to cover a broader population and demonstrated greater data stability (31). This suggests that sample size may not only influence the reliability of research findings but could also contribute to potential misinterpretation of the prevalence of oral frailty among patients with cancer.

Given the high heterogeneity in the pooled prevalence of oral frailty in this study, the findings regarding prevalence should be interpreted with caution. Furthermore, because the cancer patients included in this study did not cover all cancer types, the pooled prevalence cannot be directly extrapolated to patients with all cancer types or treatment stages.

Notably, all studies included in the present review were conducted in China and Japan. This geographical concentration may primarily reflect the current development of the evidence base. The concept of oral frailty was originally proposed in Japan and was subsequently operationalized through screening instruments such as the Oral Frailty Index-8 and the Oral Frailty Five-item Checklist (32–34). Accordingly, much of the early conceptual, methodological, and epidemiological research on oral frailty has been conducted in Japan, followed by an increasing number of studies from China and other East Asian populations. Although recent cross-cultural adaptation studies indicate growing interest in oral frailty outside East Asia, evidence specifically concerning patients with cancer remains scarce in other regions. In addition, previous conceptual reviews have demonstrated variation in the definitions and operationalization of oral frailty (35). Although our search strategy incorporated both controlled vocabulary and free-text terms related to oral frailty and oral function, variations in terminology across regions may partly explain the observed geographical concentration of available evidence.

### Potential mechanisms underlying the influencing factors of oral frailty in patients with cancer

Denture use was statistically associated with the development of oral frailty in patients with cancer, which is consistent with previous findings (36). This study demonstrated an association between denture use and oral frailty (OR = 15.09). However, this effect size is markedly higher than that reported in previous studies and should therefore be interpreted with caution. First, the number of studies included in this analysis was limited (only two studies), which may have resulted in unstable estimates and an inflation of the effect size. Denture base materials, particularly polymethyl methacrylate (PMMA) acrylic resin, possess surface characteristics that facilitate the formation of plaque biofilms, thereby serving as reservoirs for *Candida* species and bacteria. Evidence has demonstrated that the adhesion and biofilm formation of *Candida albicans* on PMMA surfaces are major contributors to the development of denture stomatitis (37). Candida infection and the associated local inflammatory response can lead to the release of a large number of inflammatory mediators, which not only compromise the integrity and reparative capacity of the oral mucosa but may also induce a state of low-grade systemic inflammation (38). This chronic inflammation may affect oral health (39, 40).

Pooled analyses suggested association between a history of radiotherapy and oral frailty; a similar association was observed for xerostomia. A potential mechanistic hypothesis may be derived from basic research, in which radiation may induce DNA damage and activation of autophagic pathways in salivary gland cells, potentially leading to a reduction in functional acinar cells and impaired glandular regenerative capacity (41). In addition, radiation disrupts metabolic homeostasis and neural regulation of the salivary glands. Recent histological and metabolomic analyses have demonstrated that radiation leads to metabolic reprogramming in salivary gland cells, characterized by decreased glycolysis and mitochondrial oxidative phosphorylation, along with the accumulation of amino acid metabolites. This metabolic dysregulation not only compromises energy supply to the glandular tissue but may also impair membrane transport and secretory mechanisms, thereby further reducing salivary production and secretion (42). In summary, these mechanisms may contribute to reduction in salivary flow following radiotherapy. Saliva plays a critical role in antimicrobial defense, antiviral activity, and protection of the oral mucosa. Reduced salivary secretion may compromise this natural protective barrier, potentially facilitating bacterial and fungal colonization and increasing the risk of infection (43). Persistent xerostomia may also lead to degenerative changes in the oral mucosa and increase the incidence of oral ulcers, gingivitis, and other conditions, thereby further worsening oral health status (44).

Physical frailty and malnutrition appeared to have a moderate statistical association with oral frailty in patients with cancer. This is consistent with the findings reported by Chen et al. (45). From the perspective of muscle metabolism, both conditions can induce anabolic resistance, a state in which the physiological response to nutritional stimuli is blunted. This leads to downregulation of the IGF-1/PI3K/Akt/mTOR signaling pathway, thereby inhibiting muscle protein synthesis (46). Concurrently, the myostatin/activin signaling pathway is upregulated, promoting activation of the ubiquitin–proteasome system and enhancing muscle protein degradation (47). This imbalance may not only affect the skeletal muscles of the limbs but may also involve the lingual muscles, masticatory muscles, and swallowing-related muscle groups, manifesting as reduced tongue pressure, decreased bite force, and impaired swallowing function (48, 49).

Studies have suggested a statistically positive association between smoking and oral frailty. From a biological perspective, evidence indicates that smokers exhibit an increased abundance of anaerobic pathogenic bacteria and other periodontitis-associated microbial taxa, accompanied by a relative depletion of health-associated commensal species, thereby creating a pathogen-dominated oral ecosystem. This shift in microbial composition further promotes the development of periodontitis and chronic inflammation of the oral mucosa (50). Pathogenic microorganisms can disrupt the oral epithelial barrier through the production of proteolytic enzymes, virulence factors, and metabolic byproducts, compromising mucosal integrity and interfering with local immune homeostasis, which maybe lead to persistent tissue damage (51). Meanwhile, smoking can also alter the oral environment—such as by reducing oxygen tension and modifying PH levels—thereby creating favorable conditions for the proliferation of anaerobic bacteria and exacerbating microbial imbalance (52). However, due to limitations in the design of the included studies, causality cannot be inferred. A history of smoking may serve as a simple screening indicator for the early identification of individuals at high risk of oral frailty. Nevertheless, whether smoking directly leads to oral frailty remains to be verified by prospective cohort studies.

Given that the majority of included studies were of moderate quality, the observed associations should be interpreted with caution. Studies with higher risk of bias may have overestimated the effect sizes due to inadequate control of confounding factors. It should be noted that the pooled estimates for several associated factors were based on a limited number of studies: denture use and radiotherapy were each derived from only two studies, while smoking, xerostomia, and malnutrition were each based on three studies. The exceptionally wide confidence interval observed for denture use (OR = 15.09, 95% CI: 6.78–33.58) reflects considerable uncertainty around this estimate. Therefore, although these factors showed statistically significant associations, the certainty and stability of these pooled estimates are limited, and these findings should be interpreted with caution.

## Conclusion

The present study suggests a prevalence of oral frailty of 52% in patients with cancer, underscoring the need of implementing preventive interventions in this population. The analyses identified smoking, xerostomia, denture use, impaired oral health status, physical frailty, malnutrition, and radiotherapy as factors associated with oral frailty in patients with cancer. Given that all included studies were cross-sectional in design and substantial heterogeneity was observed across studies, the aforementioned associations cannot be interpreted as causal relationships. Large-scale, prospective studies employing standardized assessment tools are warranted to further validate these findings.

## Limitations

This study has several limitations that should be acknowledged. First, all included studies were cross-sectional in design, which precludes the determination of temporal sequences and causal relationships between variables. Therefore, the findings of this study can only suggest associations rather than causality. Second, the number of included studies was relatively small (*n* = 13), and most were conducted in China and Japan, indicating a geographically concentrated sample. This may limit the generalizability of the findings to other populations. Furthermore, variations in the definition, measurement tools, and diagnostic criteria for oral frailty across studies may have introduced measurement bias and contributed to between-study heterogeneity. Future studies should adopt validated, standardized diagnostic criteria for oral frailty. Third, the high heterogeneity in the pooled prevalence of oral frailty in patients with cancer suggests the presence of unmeasured or unreported confounding factors, such as treatment stage and baseline health status. These variables were inconsistently reported in the original studies, limiting the possibility of more in-depth analyses. Finally, only articles published in Chinese and English were included, which may have introduced retrieval bias. Future research should consider including studies published in additional languages to further validate the conclusions of this study. In summary, future studies should focus on large-scale, multicenter, prospective designs using standardized oral frailty assessment tools, and should perform detailed analyses across different cancer types and treatment regimens to further validate these findings and improve the overall quality of evidence. Moreover, future research should systematically report details such as cancer type, TNM stage, specific treatment protocols, and baseline status to facilitate more refined subgroup analyses and meta-regression.

## Data Availability

The original contributions presented in the study are included in the article/Supplementary material, further inquiries can be directed to the corresponding author.
