# Supplementary material for: Prevalence and influencing factors of oral frailty in cancer patients: a systematic review and meta-analysis
Source: Front Public Health. 2026 Aug 24;14:1859081. doi: 10.3389/fpubh.2026.1859081 (PMC13547065; doi:10.3389/fpubh.2026.1859081)
Supplement: Supplementary file 1 [file Table_1.docx]

**Table S1 The search strategy**

| Database | Query | Results |
| --- | --- | --- |
| PubMed | #1：（"oral function"[MeSH Terms]) OR ("oral weakness"[Title/Abstract] OR "oral frail*"[Title/Abstract]) | 284 |
|  | #2：(Neoplasms[MeSH Terms]) OR (Neoplasm*[Title/Abstract] OR Tumor*[Title/Abstract] OR Neoplasia*[Title/Abstract] OR Cancer*[Title/Abstract] OR Malignancy[Title/Abstract] OR Malignancies[Title/Abstract]) | 5513412 |
|  | #1 and #2 | 16 |
| WOS | #1：TI=(“oral function” or “oral weakness” or “oral frail*” ) | 3045 |
|  | #2：TI=(Tumor* or Neoplasia* or Neoplasm or Cancer* or Malignancy or Malignancies ) | 10313038 |
|  | #1 and #2 | 518 |
| EMBASE | #1：'oral function':ab,ti OR 'oral function':ab,ti OR 'oral weakness':ab,ti OR 'oral frail*':ab,ti | 3174 |
|  | #2：'tumor':ab,ti OR 'neoplasia':ab,ti OR 'neoplasm':ab,ti OR 'cancer':ab,ti OR 'malignancy':ab,ti OR 'malignancies':ab,ti | 8265182 |
|  | #1 and #2 | 574 |
| Cochrane Library | #1：(“oral function” OR “oral weakness” OR “oral frailty” OR "oral frail"):ti,ab,kw | 423 |
|  | #2：(Tumor* or Neoplasia* or Neoplasm or Cancer* or Malignancy or Malignancies):ti,ab,kw | 301991 |
|  | #1 and #2 | 43 |
| CINAHL | TX (“oral function” OR “oral weakness” OR "oral frail*") AND TX (Tumor* or Neoplasia* or Neoplasm or Cancer* or Malignancy or Malignancies) | 87 |
| VIP | #1：M=(口腔衰弱 or 口腔功能) | 365 |
|  | #2:R=(癌症 or 肿瘤) | 1052067 |
|  | #1 and #2 | 21 |
| sinomed | #1：“口腔衰弱”[全部字段:智能] | 172 |
|  | #2：(“癌症”[常用字段:智能] OR “肿瘤”[常用字段:智能]) | 2213236 |
|  | (#1) AND (#2) | 5 |
| Wanfang | 主题:(口腔衰弱) and 题名或关键词:(癌症 or 肿瘤) | 13 |
| CNKI | （主题：口腔衰弱）AND（篇关摘：癌症 + 肿瘤（精确）） | 5 |
