# Supplementary material for: Prevalence and influencing factors of oral frailty in cancer patients: a systematic review and meta-analysis
Source: Front Public Health. 2026 Aug 24;14:1859081. doi: 10.3389/fpubh.2026.1859081 (PMC13547065; doi:10.3389/fpubh.2026.1859081)
Supplement: Supplementary file 2 [file Table_2.docx]

**Table S2**

| Author | #1 | #2 | #3 | #4 | #5 | #6 | #7 | #8 | #9 | #10 | #11 | Total Score |
| --- | --- | --- | --- | --- | --- | --- | --- | --- | --- | --- | --- | --- |
| Utae Katsushima | 1 | 1 | 1 | 1 | 0 | 0 | 1 | 1 | 1 | 0 | 1 | 8 |
| Fangyi Li | 1 | 1 | 1 | 0 | 0 | 1 | 1 | 1 | 0 | 1 | 1 | 8 |
| Ryugo Teranishi | 1 | 1 | 1 | 1 | 0 | 0 | 1 | 1 | 0 | 0 | 0 | 6 |
| Xiaoyu Wang | 1 | 1 | 1 | 0 | 0 | 1 | 1 | 1 | 1 | 1 | 0 | 8 |
| Tao Liu | 1 | 1 | 1 | 0 | 0 | 1 | 0 | 1 | 0 | 0 | 0 | 5 |
| Liu Lijuan | 1 | 1 | 1 | 0 | 0 | 1 | 1 | 1 | 0 | 1 | 0 | 7 |
| Lu Chengqian | 1 | 1 | 1 | 0 | 0 | 1 | 1 | 1 | 0 | 1 | 0 | 7 |
| Su Rong | 1 | 1 | 1 | 0 | 0 | 1 | 1 | 1 | 0 | 1 | 0 | 7 |
| Li Yi | 1 | 1 | 1 | 0 | 0 | 1 | 1 | 1 | 0 | 1 | 0 | 7 |
| Bu Xiaolan | 1 | 1 | 1 | 0 | 0 | 1 | 1 | 1 | 0 | 1 | 0 | 7 |
| Xiaoying Shen | 1 | 1 | 1 | 1 | 0 | 1 | 0 | 1 | 0 | 1 | 0 | 6 |
| Jingwen Lv | 1 | 1 | 1 | 0 | 0 | 1 | 1 | 1 | 1 | 1 | 0 | 8 |
| Yutaro Shimizu | 1 | 1 | 1 | 0 | 0 | 0 | 1 | 1 | 1 | 1 | 0 | 7 |

Explanations

#1：Define the source of information (survey, record review)

#2：List inclusion and exclusion criteria for exposed and unexposed subjects (cases and controls) or refer to previous publications

#3：Indicate the time period used for identifying patients

#4：Indicate whether or not subjects were consecutive if not population-based

#5：Indicate if evaluators of subjective components of the study were masked to other aspects of the status of the participants

#6：Describe any assessments undertaken for quality assurance purposes (e.g., test/retest of primary outcome measurements)

#7：Explain any patient exclusions from analysis

#8：Describe how confounding was assessed and/or controlled

#9：If applicable, explain how missing data were handled in the analysis

#10：Summarize patient response rates and completeness of data collection

#11：Clarify what follow-up, if any, was expected and the percentage of patients for which incomplete data or follow-up was obtained

**Table S3 Potential confounders and covariates included in the multivariate analysis**

| Study | Confounders |
| --- | --- |
| Utae Katsushima |  |
| Fangyi Li | age, educational level, disease duration, number of chronic diseases, history of radiation therapy, dry mouth, and denture use, chemotherapy-induced taste alteration, acceptance of illness, family functioning |
| Ryugo Teranishi |  |
| Xiaoyu Wang | marry status, education , income, smoking history, denture use, Xerostomia, physical frailty, oral health, nutritional risk, platelet to lymphocyte ratio, neutrophil to lymphocyte ratio |
| Xiaoying Shen |  |
| Yutaro Shimizu | age, smoking history, hemoglobin , albumin , mini nutritional assessment – short form |
| Tao Liu | dietary diversity score, smoking |
| Liu Lijuan | age, history of lung surgery, chronic obstructive pulmonary disease, educational level, tumor staging, presence or absence of brain metastases, history of cranial radiotherapy, nutritional risk screening 2002 score, pain score, fatigue score, hemoglobin, albumin, C-reactive protein, neutrophil to lymphocyte ratio, platelet to lymphocyte ratio, lactate dehydrogenase |
| Lu Chengqian | age, marry status, educational level, radiotherapy, chemotherapy, grip, oral mucositis, Poor oral health, oral self-efficacy score, nutritional risk |
| Su Rong | age, smoking history, nasogastric intubation, Xerostomia, physical frailty, nutritional level, poor oral health, anxiety, depression, grip, number of remaining natural teeth |
| Li Yi | gender, age, smoking history, chemotherapy history, number of chronic diseases, Xerostomia, denture use, poor oral health, nutritional risk, physical frailty |
| Bu Xiaolan | age, number of children, educational level, occupational category, monthly income, tumor staging, cancer-related anorexia, family functioning, chemotherapy-induced taste alteration, insomnia, anxiety, depression |
| Jingwen Lv | radiotherapy history, tumor staging, physical frailty, smoking history, age, nutritional status |
