# Supplementary material for: Prevalence and influencing factors of oral frailty in cancer patients: a systematic review and meta-analysis
Source: Front Public Health. 2026 Aug 24;14:1859081. doi: 10.3389/fpubh.2026.1859081 (PMC13547065; doi:10.3389/fpubh.2026.1859081)
Supplement: Supplementary file 3 [file Table_3.docx]

**Additional File 3.**

**1. Forest Plot of Subgroup Analysis of Influencing Factors for Oral Frailty in Cancer Patients**

**1.1 Age**

Fig.1. Forest plot of influencing factors of age.

**1.2 Smoking**

Fig.2. Forest plot of influencing factors of smoking.

**1.3 Dry mouth**

Fig.3. Forest plot of influencing factors of dry mouth.

**1.4 Denture use**

Fig.4. Forest plot of influencing factors of denture use.

**1.5 Poor oral health**

Fig.5. Forest plot of influencing factors of poor oral health.

**1.6 Physical frailty**

Fig.6. Forest plot of influencing factors of Physical frailty.

**1.7 Nutritional risk**

Fig.7. Forest plot of influencing factors of nutritional risk.

**1.8 Depression**

Fig.8. Forest plot of influencing factors of depression.

**1.9 Radiotherapy**

Fig.9. Forest plot of influencing factors of radiotherapy.

**2. Forest Plot of Subgroup Analysis for the Prevalence of Oral Frailty in Cancer Patients**

**2.1 Gender**

Fig.10. Forest plot of prevalence by gender

**2.2 Country**

Fig.11. Forest plot of prevalence by country

**2.3 Sample Size**

Fig.12. Forest plot of prevalence by sample size

**2.4 Measurement Tool**

Fig.13. Forest plot of prevalence by measurement tool

**2.5 Study Centers**

Fig.14. Forest plot of prevalence by number of study centers

**2.6 TNM Stage**

Fig.15. Forest plot of prevalence by TNM stage

**2.7 Cancer type**

Fig.16. Forest plot of prevalence by cancer type

**3.Publication of bias analysis graphs**

Fig.17. Funnel plot for publication bias.

Fig.18. Egger's linear regression test for publication bias
